# Modelling clinical trial recruitment using poisson processes

**DOI:** 10.1186/1745-6215-16-S2-P85

**Published:** 2015-11-16

**Authors:** Kristian Brock, Christina Yap, Gary Middleton, Lucinda Billingham

**Affiliations:** 1 Cancer Research UK Clinical Trials Unit, University of Birmingham, Birmingham, UK; 2 School of Cancer Sciences, University of Birmingham, Birmingham, UK

## 

Forecasting recruitment to clinical trials is often too simplistic and rarely statistical. This is unfortunate because modelling trial recruitment is simple using Poisson processes. The simplest case is to assume patients arrive randomly at a constant rate, using homogeneous Poisson processes to simulate sample recruitment curves. In contrast, time-varying complexity can be incorporated using non-homogeneous Poisson processes. This necessitates a recruitment intensity function, 0 ≤ p(t) ≤1, (t ≥ 0), to describe the instantaneous rate of recruitment potential at time t, as a proportion of the maximum rate possible.

Using this method, one can incorporate important factors like seasonal trends, staggered opening of recruitment centres, accelerating and decelerating recruitment and planned suspensions (for instance, due to interim assessments), to reflect more accurately the possible recruitment patterns over time.

There are many benefits to investing the time to model trial recruitment. Guidance on plausible recruitment scenarios can inform inventory needs and forewarn the impact of recruitment shocks, both welcome and unwelcome. In designs with time-to-event outcomes, the rate of patient recruitment can affect statistical operating characteristics. In complicated trial designs, it may be especially important to model recruitment to demonstrate trial feasibility.

We present the mathematics of the homogeneous and non-homogeneous cases, along with simple, versatile algorithms for simulating each using open-source R code. We discuss the insights we garnered by modelling recruitment to the National Lung Matrix trial, a multi-arm, multi-drug, phase II adaptive design stratified by random genetic changes in non-small cell lung cancer patients.

